# Biological Activity of Biosynthesized Silver Nanoaggregates Prepared from the Aqueous Extract of *Cymbopogon citratus* against *Candida* spp.

**DOI:** 10.3390/nano13152198

**Published:** 2023-07-28

**Authors:** Fatimah Al-Otibi, Luluwah S. Albulayhid, Raedah I. Alharbi, Atheer A. Almohsen, Ghada M. AlShowiman

**Affiliations:** Department of Botany and Microbiology, College of Science, King Saud University, Riyadh 11495, Saudi Arabia; lulusul09@gmail.com (L.S.A.); raalharbi@ksu.edu.sa (R.I.A.); atheer.ab.almohsen@gmail.com (A.A.A.); gshowinan@ksu.edu.sa (G.M.A.)

**Keywords:** *Cymbopogon citratus*, silver nanoparticles, anti-candida, SEM, TEM, FTIR

## Abstract

*Cymbopogon citratus* is commonly used in folk medicine for the treatment of nervous and gastrointestinal disturbances and other medical issues because of its potent antioxidant capacity. The current study evaluated the anti-candida effects of silver nanoparticles (AgNPs) synthesized from an aqueous extract of *C. citratus* against different *Candida* spp. The aqueous extract was prepared from the fresh leaves of *C. citratus*. The silver nanoparticles (AgNPs) were prepared and validated by UV spectroscopy, Fourier-transform infrared spectroscopy (FTIR), transmission electron microscope (TEM), and zeta size analysis. *C. albicans*, *C. krusei*, *C. parapsilosis*, *C. tropicalis*, *C. famata*, *C. rhodotorula*, and *C. glabrata* were used in the antifungal assay. Microscopical imaging were used to investigate the different morphological changes induced by treatment. FTIR spectrum confirmed the existence of various functional groups of biomolecules capping the nanoparticles. The average particle size of synthesized AgNPs was 100.6 nm by zeta-sizer and 0.012 to 0.059 mm by TEM. In the antifungal assay, AgNPs aggregates induced significant inhibition of the growth of all species (*p* < 0.05) compared to the control and the biofilm maturation in *C. famata* and *C. albicans*. These considerable antifungal activities might lead to the development of appropriate alternative remedy for the treatment of fungal infections.

## 1. Introduction

Silver nanoparticles (AgNPs) are particularly appealing and fascinating for a variety of applications because of their unique and amazing features, improved permeability, retention effect, and antibacterial activity [1]. AgNPs are widely employed because of its broad antibacterial action against a variety of microbes and localized surface plasmon resonance impact [2]. 

There are few thoughts about the mechanisms of AgNPs’ antibacterial activities. One of these mechanisms is that AgNPs can disrupt the membrane of the bacterial cell [2,3,4]. AgNPs entered the bacterial cells, causing damage to organelles, such as mitochondria and ribosomes, which resulted in chromatin condensation and margination, a sign of apoptotic cell death [5,6]. Furthermore, due to their small size, AgNPs may adhere to the cell surface and enter the cells without destroying the cell wall or causing cell death [1,7]. Another theory included oxidative stress and reactive oxygen species (ROS) production by silver ions, which causes the inactivation of several cellular proteins and activates apoptosis pathways [8]. Furthermore, there are some thoughts that, after the entrance of silver ions, they might bind to the phosphoric acid residues or N7 atom of guanine in DNA molecules, which affects its replication and cellular division [6].

The biological synthesis of silver nanoparticles, also known as green synthesis, is a preferable method of preparation because it avoids the drawbacks of other chemical or physical techniques, such as high costs, higher temperatures, and the production of waste and hazards [6]. On the other hand, the green synthesis of nanoparticles using plant extracts mixed with silver oxide or silver nitrate has the advantages of low cost, low toxicity, and high antimicrobial activities, which further enables their uses in biomedicine [9]. 

The interest in medicinal plants is growing significantly in both developed and developing nations’ primary healthcare systems [10]. The ancient practice of utilizing plant preparations to battle fungal diseases has gained popularity, and the present focus is on the discovery of novel antifungal components from plants that have no harmful impact on the environment, animal, or human systems [11]. 

Lemongrass, *Cymbopogon citratus* (also known as stapf, Cochin grass, or Malabar grass), is a tropical plant produced in nations such as India, Bangladesh, Oman, Yemen, Sri Lanka, Burma, Indonesia, and Saudi Arabia [12]. It belongs to the *Poaceae* or *Gramineae* family, which has about 700 genera and over 1200 species of monocotyledonous flowering plants [13]. *C. citratus* is widely used in folk medicine to treat mental and gastrointestinal disorders, as well as an antispasmodic, analgesic, anti-inflammatory, anti-pyretic, diuretic, antioxidant, and sedative [14]. Decoctions of *C. citratus* leaves and petals are used to treat skin problems, conjunctivitis, migraine, and hepatitis; also, essential oils (EO) produced from that herb are a typical drug used in aromatherapy [15].

In vitro, distinct *Gramineae* family members have demonstrated varying medicinal characteristics. *Cymbopogon martini* and *C. citratus* demonstrated considerable anthelmintic action, which was assumed to be due to the phenolic concentration of geraniol [16]. *C. citratus*-induced apoptosis, DNA fragmentation, and caspase-3 catalytic activity in cervical, breast, and prostate cancer cells [17,18]. The anti-inflammatory activities of lemongrass EO were investigated in a pharmacological investigation in mice using 5 µL of lemongrass oil per mouse intraperitoneally [19]. Lemongrass oil has been proven to limit neutrophil accumulation and thereby reduce leukocyte migration into the peritoneal cavity. In another research, 0.0125 to 0.1% lemongrass oil inhibited TNFα-induced neutrophil activation in vitro [18]. Another study used *C. citratus* EO to treat pre-inflamed human dermal fibroblasts and discovered that it might impact the production of many inflammatory markers such as VCAM-1, IP-10, I-TAC, MIG, collagen-I and III, EGFR, PAI-1, and M-CSF [20]. The vapor phase of EOs extracted from *C. martinii* demonstrated strong antifungal activity against *Penicillium expansum*, *Fusarium graminearum*, and *Candida albicans*, which was thought to be due to K^+^ leakage from the fungal cell membrane, which caused serious ultrastructural deformations and subsequently affected fungal growth [8,17,21]. Non-EO extracts of *C. citratus* were limitedly tested for antifungal activity against *Aspergillus niger* [22] and *Candida albicans* [23]. 

These constraints reinforced the necessity for more analytical investigations of the anti-candida activities of C. citratus non-EO extracts against different *Candida* species. The current work focused on exploring the anti-candida functions of the aqueous extract and generated AgNPs from *C. citratus* leaves against several *Candida* spp.

## 2. Materials and Methods

### 2.1. Study Design

In the current study, the toxic fungicidal effects of the aqueous extract of *C. citratus* against the planktonic cells of seven Candida species were evaluated by calculating the minimal inhibitory concentration (MIC) from the measures of the zones of inhibition (ZOI). Also, the antifungal properties of the AgNPs synthesized from the aqueous extract of *C. citratus* were assessed against all species and compared to the negative and positive controls. Furthermore, the scanning electron microscope (SEM) was used to investigate any possible ultrastructural changes in the proliferation of the plasmalemma and thickness of the cell membrane. 

### 2.2. Vegetal material

The fresh leaves of *C. citratus* were collected from the farm located at Ad Diriyah region (PJP9 + RM) King Saud University, Riyadh, Saudi Arabia. The leaves were collected during the summer season of 2022. 

The fresh leaves were washed with fast flowing water, followed by continuous rinsing with deionized water for 10–15 min to remove any possible contaminants of dust or other impurities. The leaves were dried in a controlled greenhouse by air circulation at 37 °C for 48 h. In total, 250 g of the dried leaves were then grinded in a cutting mill, collected in a sterile glass container and stored at 4 °C until use. 

To prepare the aqueous extract of *C. citratus* leaves, the powdered leaves were immersed in 350 mL of boiling deionized water for 30–60 min. The liquid supernatant was collected by filtration through tissue papers (Sartolab^®^ RF Vacuum Filtration Units 180C8, Goettingen, Germany), where the process was repeated four times. At the end, the filtrate was collected and stored at 4 °C until use.

### 2.3. Microbial Strains

Seven Candida strains were used to assess the antifungal activity of *C. citratus*. The species were obtained from the American Type Culture Collection (ATCC, Manassas, VA, United States) and included *C. albicans* (ATCC^®^ 90028™), *C. krusei* (ATCC^®^ 32196™), *C. parapsilosis* (ATCC^®^ 28474™), *C. tropicalis* (ATCC^®^ 18807™), *C. famata* (ATCC^®^ 10619™), *Rhodotorula* sp. (ATCC^®^ 9449™), and *C. glabrata* (ATCC^®^ 90030™). 

Candida strains were cultured in a potato dextrose agar (PDA) medium. Briefly, 33.5 g of PDA were mixed with distilled water, boiled until dissolve, then cooled to 45–50 °C. After cooling, the agar was aliquoted into sterile Petri dishes at the thickness of 5 mm and left to dry at room temperature in a sterile safety cabinet. 

The studied strains were cultured on PDA Petri dishes at 28 °C for 48 h, as previously described [24]. The turbidity of growing Candida suspension was adjusted to match the turbidity standard of 0.5 McFarland units, by spectrophotometry at 530 nm (O.D. of 0.12 is equal to 1 × 10^6^ CFU/mL) [25]. 

### 2.4. Green Synthesis of AgNPs

AgNPs were synthesized from the aqueous extract of *C. citratus*, as described before [26], with some modifications as shown in Supplementary Figure S1. Briefly, 10 mL of the prepared extract was mixed with 100 mL of AgNO_3_ (2 mM) solution. The reaction was kept at room temperature for less than 1 h, during which a color change from colorless to transparent yellow and finally dark brown was observed. That indicated the reduction of Ag^+^ ions in the alkaline mixture the aqueous extract of *C. citratus* at pH of 9.52. The biosynthesized AgNPs were purified by the PD-10 desalting column (Sigma-Aldrich, St. Louis, MI, USA), as described before [27].

### 2.5. Characterization of AgNPs

The formation and characterization of the synthesized AgNPs were analyzed against either the Crude extract of *C. citratus* or AgNO_3_, as follows:

#### 2.5.1. UV-Visible Spectroscopy

UV-visible spectrophotometer UV-2450 double-beam (Shimadzu, Tokyo, Japan) was used for the optical study of the synthesized AgNPs by measuring the attenuation of light passes through the irradiated particles. The reduction of AgNPs by the alkaline aqueous extract (pH of 9.52) was assessed at 300–800 nm after 1 h exposure at 40 °C, according to the manufacturer’s guidelines.

#### 2.5.2. Fourier-Transform Infrared Spectroscopy (FTIR)

FTIR spectroscopy was carried out to identify the biomolecules that bound specifically on the silver surface and local molecular environment of capping agent on AgNPs. At the beginning, the prepared AgNPs colloidal solution was centrifuged at 5000 rpm for 10 min, to remove any insoluble residues or compounds. Then, the supernatant was recentrifuged at 10,000 rpm for 60 min to pellet the synthesized AgNPs. The obtained pellet was analyzed by Nicolet—6700 spectrometer (Thermo Fisher Scientific Inc., Waltham, MS, USA). The results were expressed as peaks in the range of 500–4000 cm^−1^ and the functional groups were interpreted using the guidelines of LibreText’s libraries https://chem.libretexts.org/ (accessed on 1 February 2023) [28].

#### 2.5.3. Dynamic Light Scattering (DLS) and Zeta-Potential

DLS and zeta-potential are useful tools to assess the physicochemical properties of the nanoparticles, besides evaluation of their size, stability, and surface charge [29]. In the current study, the Zetasizer Pro (Malvern Panalytical, Malvern, UK) was used as been described before [27]. The values of polydispersity index (PDI) and Z-average size were used to calculate the PDI width (z-average × PDI).

#### 2.5.4. Transmission Electron Microscope (TEM)

The purified AgNPs were analyzed by TEM imaging to better describe the physical characteristics of nanoparticles, such as size and morphology, as it enables higher resolution than other imaging facilities. In the current study, nanoparticles were analyzed by a JEM-1400 transmission electron microscope (JEOL Ltd., Tokyo, Japan), according to the manufacturer’s instructions [27].

### 2.6. Antifungal Properties of C. citratus Aqueous Extract and Synthesized AgNPs

The antifungal activities of biosynthesized AgNPs and the aqueous extract of *C. citratus* against tested species were evaluated by the agar well-diffusion method [30]. Using a sterile metallic cork borer, four holes of 6–8 mm were punched in each culture Petri dish containing one of the studied species. Four different treatments (15 µL each) were separately added in one of the wells in each dish, which included either 10% of the aqueous extract of *C. citratus* (prepared in distilled water), 100% of the synthesized AgNPs, AgNO_3_ (2 µM), or 0.125 μg/mL of Terbinafine (as positive control). The plates were incubated at 28 °C for 48 h. The diameter of zones of inhibitions (ZOI) of each treatment were measured in mm and compared to control. The growth areas of different treatment were expressed by subtracting the diameter of the ZOIs from the total growth area (mm). The minor changes in the ZOI diameter were calculated using ImageJ software available from https://imagej.nih.gov/ij/download.html (accessed on 22 February 2023). The experiment was performed in triplicates.

### 2.7. Imaging of the Strains Mostly Affected by C. citratus Aqueous Extract and Synthesized AgNPs

To evaluate and assess all possible ultrastructural changes of the *Candida* strains, which were excessively affected by *C. citratus* aqueous extract and synthesized AgNPs, TEM and a scanning electron microscope (SEM) were used. Both imaging techniques were used. The methodology of the TEM was described above. For the SEM, samples were prepared and analyzed in the Central Laboratory at the Women Students’ Medical Studies and Sciences Sections, King Saud University, Riyadh, Saudi Arabia, according to their procedure.

### 2.8. Statistical Analysis

The results were analyzed using the IBM^®^ SPSS software. All experiments were performed in triplicates, where the mean values and standard deviation (SD) were calculated for ZOI diameters. One-way analysis of variance (ANOVA) was used to assess the statistical significance, which was set at *p* < 0.05.

## 3. Results

### 3.1. Successful Synthesis and Characterization of AgNPs the Aqueous Extract of C. citratus

In the current study, the synthesis of AgNPs from the aqueous extract of *C. citratus* was noted by the change in the color of AgNO_3_ from colorless to light brown (Supplementary Figure S1). After purification of the produced nanoparticles, the synthesis process was validated by UV spectroscopy, FTIR, DLS, and TEM.

**Figure 1:**
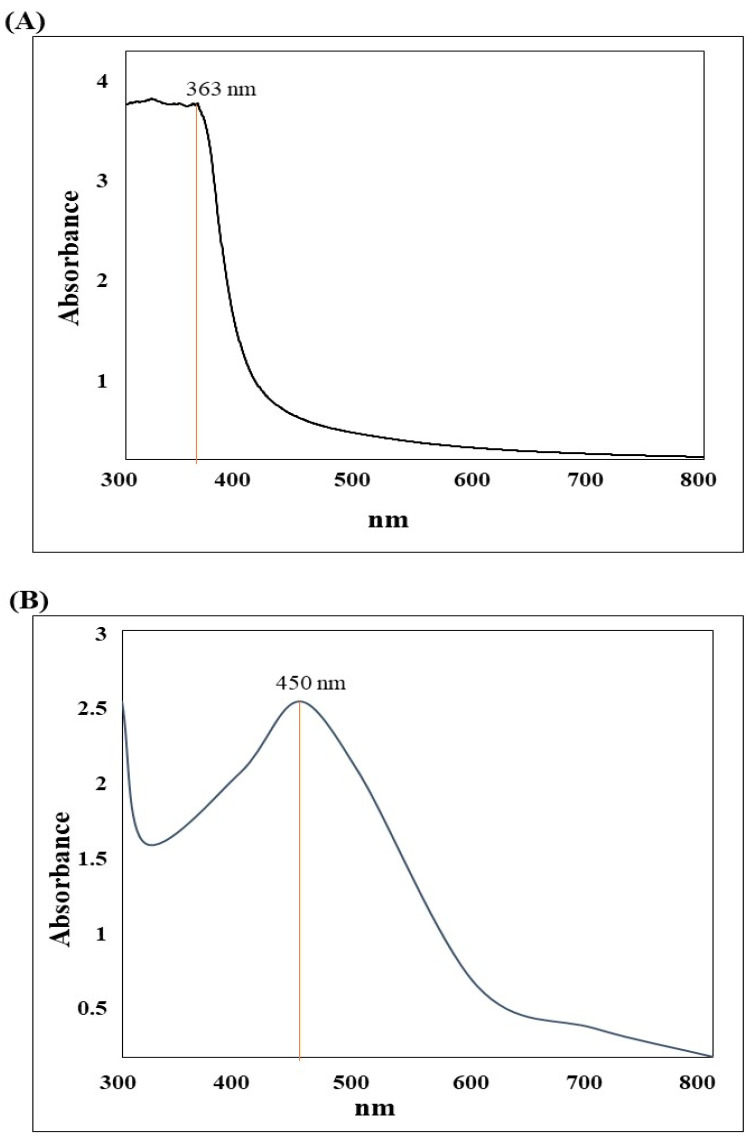
The UV–VIS spectrum of *C. citratus* products. The spectrum was analyzed by the Shimadzu UV–visible spectrophotometer, (**A**) *C. citratus* aqueous extract. (**B**) *C. citratus* AgNPs.

As shown in Figure 1, the UV spectrum analysis of the aqueous extract of *C. citratus* caused an attenuation equal to a Surface Plasmon Resonance (SPR) of 363 nm, compared to 455 nm for AgNPs. © Highest peak of AgNPs spectra was broad, smooth, well-defined, and had an absorption of the UV beam equal to 2.5 O.D. These results emphasize the formation of AgNPs.

**Figure 2:**
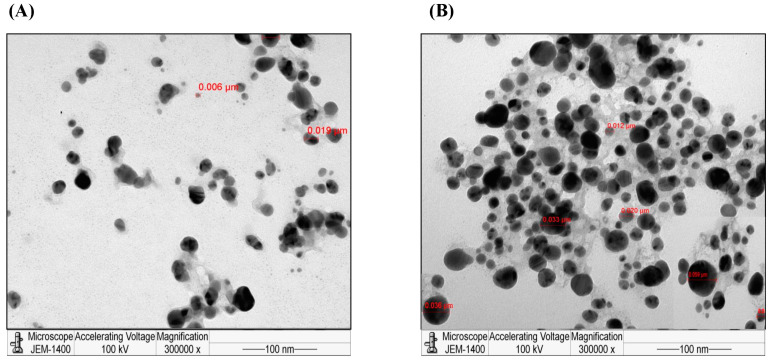
Characterization of the synthesized *C. citratus* AgNPs by TEM. The images were obtained by the JEOL JEM-1400 transmission electron microscope. (**A**) TEM image of AgNO_3_. (**B**) TEM image of *C. citratus* AgNPs.

TEM images of the *C. citratus* AgNPs showed that they had different sizes ranging from 0.012–0.059 µm as compared to the small sizes of AgNO_3_ (0.006–0.019 µm) (Figure 2). The organic compounds were located on the surface and between the AgNPs aggregates (visible as moiré).

**Figure 3:**
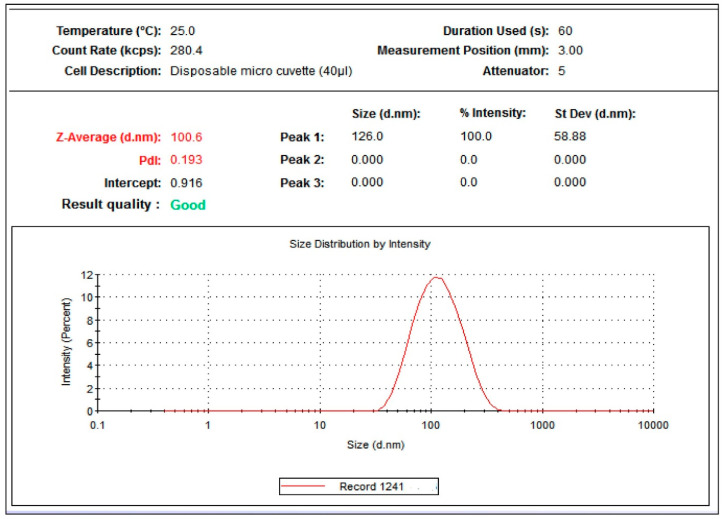
DLS analysis of *C. citratus* AgNPs. The nanoparticles were analyzed by Zetasizer Pro at 25 °C for 60 s.

The Zetasizer showed that the average particle size of *C. citratus* AgNPs aggregates was 100.6 d. nm, with a polydispersity index (PDI) value of 0.193 and an intercept of 0.916 (Figure 3). The results were based on the intensity where, at 100% intensity, the average peak size was 126 ± 58.88 d. nm. From these results, the PDI width was equal to 19.4158.

So, combing the results of UV-visible, TEM, and DLS analysis, it might be concluded that that the *C. citratus* AgNPs in the colloid were aggregated.

**Figure 4:**
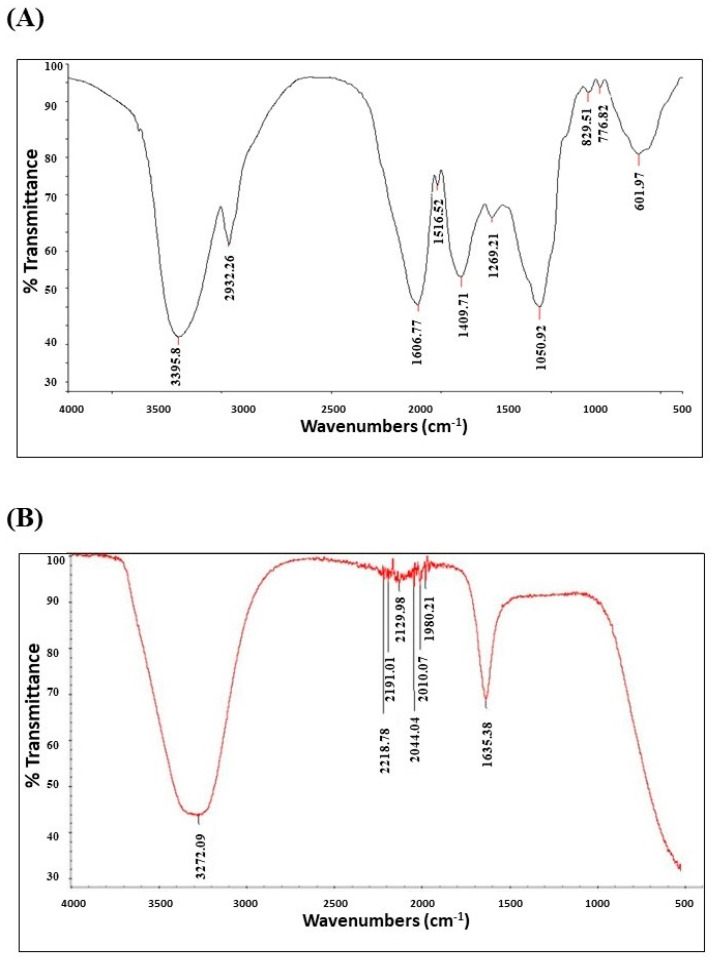
FTIR analysis of results of *C. citratus* products. The results were produced by the Nicolet 6700 FTIR Spectrometer at 500–4000 cm^-1^. (**A**) FTIR results of the aqueous extract, (**B**) FTIR results of AgNPs.

FTIR analysis resulted in eight wavenumbers, which represented the main functional groups of the *C. citratus* tested materials (Figure 4, Table 1). For the *C. citratus* aqueous extract (Figure 4B), it was shown that amines and alkenes were the most represented functional groups, followed by alcohols. For AgNPs aggregates (Figure 4B) the FTIR report showed that carboxyl groups were the most represented. Multiple carbon bonds were represented in three peaks of alkynes and three peaks of allenes. Finally, a single medium peak represented the amine group at 1635 cm^−1^.

### 3.2. The Aqueous Extract and Synthesized AgNPs of C. citratus Proved Remarkable Growth Inhibition of the Tested Candida Strains

**Figure 5:**
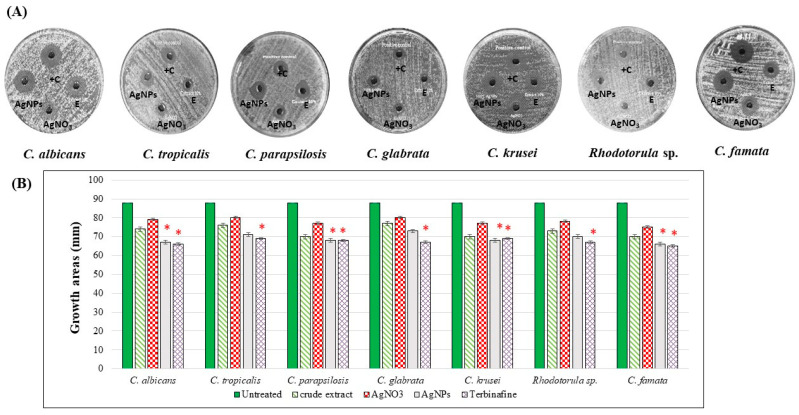
The antifungal activities of different treatments of *C. citratus*. Candida strains were grown on PDA dishes for 24 h. By the well-diffusion method, the cultured were treated by either 10% of the aqueous extract of *C. citratus*, 100% of the synthesized AgNPs, AgNO_3_ (2 µM), or 0.125 μg/mL Terbinafine. (**A**) Petri dishes of the treated species. (**B**) Chart of the growth areas diameters. E: crude extract; +C: Terbinafine positive control, * indicated significant *p*-value less than 0.05.

In the current study, the antifungal activities of the aqueous extract and synthesized AgNPs of *C. citratus* were evaluated against seven species (Figure 5, Table 2). As shown, the crude extract (10%) decreased the growth areas of all species, however, it was statistically insignificant (*p* > 0.05). Otherwise, AgNPs induced a significant reduction in the growth areas of *C. albicans*, *C. tropicalis*, *C. parapsilosis*, *C. krusei*, and *C. famata* (*p* < 0.05). The most affected species were *C. albicans* and *C. famata*, with growth areas of 66 ± 0.15 mm and 65 ± 0.08 mm, respectively, as compared to the growth areas of non-treated controls of 88 ± 0.1 mm and 88 ± 0.3 mm, respectively. So, according to the above-mentioned results, *C. albicans* and *C. famata* were used to compare the ultrastructural changes in the cellular walls as a result of the treatments with the synthesized AgNPs of *C. citratus.*

**Figure 6:**
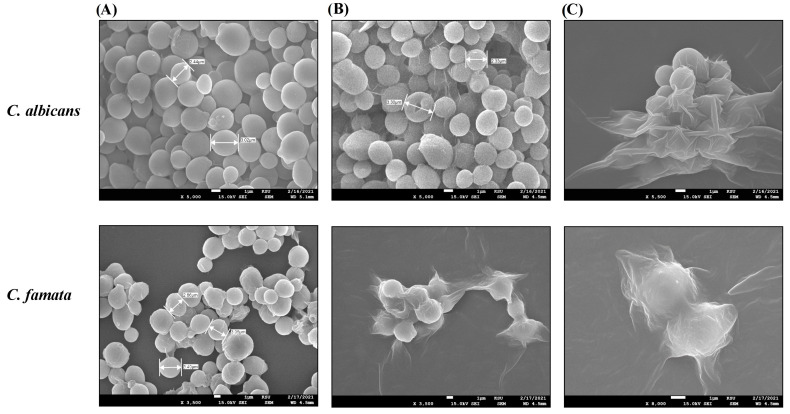
SEM images of *C. albicans* and *C. famata* treated with *C. citratus* AgNPs. SEM images were evaluated by the Scanning Electron Microscope SU3800 (HITACHI, Tokyo, Japan). (**A**) images of the untreated species at ×3500 magnification. (**B**) images of the AgNPs-treated species at ×3500 magnification for *C. famata* and ×5000 magnification for *C. albicans*. (**C**) images of the AgNPs-treated species at ×8000 magnification for *C. famata* and ×5500 magnification for *C. albicans*.

**Figure 7:**
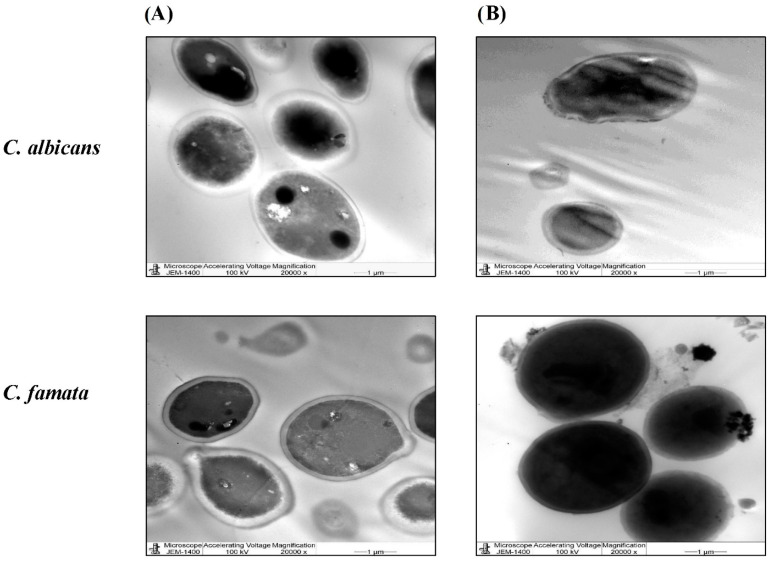
TEM images of *C. albicans* and *C. famata* treated with *C. citratus* AgNPs. TEM images were evaluated by the JEOL JEM-1400 transmission electron microscope at 20,000× magnification. (**A**) images the untreated species. (**B**) images of the AgNPs-treated species.

TEM and SEM images (Figure 6 and Figure 7) of the treated *C. albicans* and *C. famata* strains showed remarkable ultrastructural changes. As shown in Figure 6, AgNPs aggregates caused the rapture of the cell wall, where all cells did not have distinct boundaries, besides the enlargement of the cell membrane, which might be due to the uptake of AgNPs aggregates. TEM images (Figure 7) showed the weakness of the cellular membrane, which caused its rapture and release of the cytoplasm in the case of *C. albicans*, where it mostly disappeared in *C. famata*. The morphology of the *C. albicans* biofilm showed that AgNP’s aggregates had an inhibitory effect on biofilm formation, as well as fungicidal activity as the biofilm architecture was seriously damaged.

## 4. Discussion

Because of their unique and exceptional features, improved permeability, retention effect, and antibacterial activity, AgNPs are particularly appealing and fascinating for a variety of applications [31]. Silver is more poisonous to microbes than other metals, yet it is less hazardous to mammalian cells [15].

In the current study, AgNPs were synthesized from the aqueous extract of C. citratus leaves. The characterization of biosynthesized AgNPs was monitored by UV-vis spectrophotometry, DLS, and TEM. UV-vis spectroscopy showed broad absorption peak of AgNPs at 455 nm compared to 363 nm of the *C. citratus* aqueous extract. Similar to these findings, multiple studies reported the size of *C. citratus* AgNPs at 435 nm [32,33], 469 nm [26], 440 nm [34], and 450 nm [35]. These results confirmed the formation of AgNPs due to the SPR nature of AgNPs, which is directly related to their size, shape, concentration, agglomeration state, and refractive index.

DLS analysis showed that *C. citratus* AgNPs had a Z-average size of 100.6 nm and a PDI of 0.193. A previous study reported a z-average size of *C. citratus* in the range of 40–100 nm [36]. Another study reported a PDI of 0.286 and an average size of 198 nm for the AgNPs synthesized from the aqueous extract of *Cymbopogon flexuosus* [37]. Another study showed that AgNPs biosynthesized from the aqueous extract of *C. citratus* leaves had a DLS distribution of only 77.2 nm [38]. All these results indicate high-electrical charge in the AgNPs surface and has further denoted their physical stability.

Finally, the size range detected by TEM was 12–59 nm of spherical and well-dispersed AgNPs aggregates, which was larger than that of the AgNO_3_ particles. In agreement with these findings, a previous study used high-resolution TEM to screen the AgNPs synthesized from *C. flexuosus*, in which the nanoparticles appeared agglomerated with a size of 10–40 nm that was smaller than that indicated by DLS (198 nm) [37]. Other studies used TEM to analyze the *C. citratus* AgNPs sizes of 10–33 nm [32] and 5–35 nm [35]. The current findings, in accordance with other studies, prove the successful synthesis of AgNPs.

As noticed, the size detected by TEM is lower than that detected by DLS. That might be due to Brownian motion influences [39]. Also, the synthesized AgNPs aggregates were agglomerated because of the phenomenon of coagulation of the smaller particles of AgNPs [40]. Another explanation is that DLS measures the hydrodynamic radius of silver particles loaded by the bio-organic material on its surface, so the high intensity of the scattered light measured the larger (false) size of AgNPs, unlike TEM, which showed the actual size [41].

FTIR analysis of the synthesized AgNPs revealed the existence of different carboxylic, alkyne, allene, and amine functional groups. A previous study used FTIR analysis for AgNPs of *C. flexuosus* and reported bands at 3422 cm^−1^ due to the carboxyl group, 2170 cm^−1^ and 2081 cm^−1^ for alkynes, 1637 cm^−1^ for amide, 1619 cm^−1^ for carbonyl, and 1384 cm^−1^ for aldehydes [37]. Another study reported the existence of ethylene, amides, and alcohols in the FTIR results of the *C. citratus* AgNPs analysis [32]. The prominence of these groups might be due to the fact that lemongrass leaf extract is rich in terpenes, alcohols, aldehydes, ketones, esters, flavonoids, and other phenolic compounds, while the carboxylic groups are merely functioning in the direction of AgNP’s shape [42].

In recent years, severe fungal infections have significantly contributed to the increasing morbidity and mortality of immunocompromised patients who need intensive treatment, including broad-spectrum antibiotic therapy. *Candida* spp. represent one of the most common pathogens responsible for fungal infections, often causing hospital-acquired sepsis with an associated mortality rate of up to 40% [43]. Due to that, many efforts were made in the search for effective antifungal therapies that enable safety and decrease fungus resistance.

In the current study, the crude aqueous extract of *C. citratus* decreased the mycelial growth of different *Candida* spp. There are few studies that state the anti-candida properties of the aqueous extract of *C. citratus*; most of them studied the EO extract rather than any formulations. A previous study showed that the water and ethanolic extracts of *C. citratus* oils inhibited the growth of *Staphylococcus aureus*, *Staphylococcus epidermidis*, *Pseudomonas aeruginosa*, and *C. albicans* at 12.5% [44]. Multiple studies showed that the *C. citratus* EO extract revealed significant inhibition of biofilm formation of *C. tropicalis* at MIC_90_ of 2.5 µL/mL [45,46], *C. albicans* at MIC_90_ of 2.5 µL/mL [20,46], *C. krusei*, *C. parapsilosis* MIC90 of 2.5 µL/mL [46], and *C. glabrata* [46,47]. All these studies agree with the current findings about the anti-candida effects of *C. citratus*. Another study showed that the residues of ethanolic and aqueous *C. citratus* EO extracts had a significant inhibitory effect against *C. albicans* due to the phytochemical composition rich in alkaloids, flavonoids, glycosides, phenols, saponins, terpenes, tannins, fatty acids, and couarins [44]. Also, *C. citratus* EO extracts induced a ZOI diameter of 18.00–2.46 mm in the growth of *C. krusei*, which was thought to be because of its strong antioxidant activity (84%) [48].

To increase the inhibitory effects of the aqueous extract of *C. citratus*, we tested the effect of the synthesized AgNPs that were comparable to terbinafine (0.125 µg/mL). Also, AgNPs induced a more significant reduction in the growth areas of *C. albicans*, *C. tropicalis*, *C. parapsilosis*, *C. krusei*, and *C. famata*. The antifungal effect of nanoaggregates was small, but comparable to the effect of the broad-spectrum drug terbinafine. The reason for the significant anti-candida properties of AgNPs might be due to their effect on the adherence and biofilm formation of candida species, which decrease their cellular viability [49]. A previous study showed that *C. albicans* and *C. tropicalis* were highly sensitive to terbinafine (50 µg/mL), while also showing resistance to nystatin [50]. Another study showed that *C. parapsilosis*, and *C. krusei* were highly sensitive to terbinafine at 0.12 µg/mL and 4 µg/mL, respectively [51]. However, we could not find any previous studies about the anti-candida effects of biosynthesized AgNPs from the aqueous extract of *C. citratus*, although several studies stated their effect on other fungal species. A previous study used the disc-diffusion method and showed *C. citratus* EO AgNPs had a ZOI dimeter of 73.64 43.54 nm compared to *C. albicans* [52]. Another study showed that *C. citratus* EO AgNPs induced a ZOI diameter of 20.3 mm for lichens on the stone surface [34].

Finally, the microscopical images by TEM and SEM showed remarkable ultrastructural changes in *C. albicans* and *C. famata* treated with AgNPs. In accordance with these findings, a previous study showed that *C. citratus* EO AgNPs induced inhibition of *C. albicans* hyphae growth, which affected the formation of biofilms [53].

## 5. Conclusions

The current finding showed the antifungal properties of the aqueous extract of *C. citratus* leaves against some pathogenic *Candida* species. AgNPs synthesized from that extract showed robust antifungal activities similar to those of the known antifungal agent, terbinafine. That procedure provided a natural, non-toxic, economically feasible, and effective antifungal agent widespread in most areas of the tropical line, including the Kingdom of Saudi Arabia. These findings might lead to the development of appropriate alternative remedies for treating these pathogenic species, which might require further in vivo and clinical trials.

## Figures and Tables

**Table 1:** The functional group analysis by FTIR of *C. citratus* preparations.

Tested Material	Absorption (cm^−1^)	Appearance	Group	Compound Class
	3395	Medium	N-H stretching	Aliphatic primary amine
	2932	Medium	C-H stretching	Alkane
	1606	Medium	C = C stretching	Alkene
Aqueous extract	1516	Medium	N-O stretching	Nitro compound
	1409	Medium	O-H bending	Alcohol
	1269, 1050	Medium	C-N stretching	Amine
	829, 776	Strong	C-H bending	Alkene
	601	Strong	C-Br or C-I stretching	Halo-compound
	3272	Strong, Broad	O-H Stretching	Alcohol
	2218, 2191	Weak	CΞC stretching	Alkyne (disubstituted)
AgNPs	2129	Weak	CΞC stretching	Alkyne (monosubstituted)
	2044, 2010, 1980	Medium	C = C=C stretching	Allene
	1635	Medium	N-H bending	Amine

**Table 2:** The anti-candida activities of *C. citratus* (The total growth area mm).

Organisms		Negative Control	Terbinafine (0.125 µg/mL)	AgNO_3_ (2 µM)	Crude Extract (10%)	AgNPs (100%)
*C. albicans*	Mean ± SD	88 ± 0.1	74 ± 0.33	79 ± 0.23	67 ± 0.67	66 ± 0.15
	*p*-value	----------	0.019 *	0.337	0.135	0.025 *
*C. tropicalis*	Mean ± SD	87.9 ± 0.2	76 ± 0.1	80 ± 0	71 ± 0.22	69 ± 0.36
	*p*-value	----------	0.043 *	0.394	0.201	0.033 *
*C. parapsilosis*	Mean ± SD	88 ± 0.2	70 ± 0.21	77 ± 0.08	68 ± 0.01	68 ± 0.11
	*p*-value	----------	0.033 *	0.241	0.055	0.033 *
*C. glabrata*	Mean ± SD	87.8 ± 0.5	77 ± 0.35	80 ± 0.19	73 ± 0.1	67 ± 0.13
	*p*-value	----------	0.025 *	0.394	0.241	0.11
*C. krusei*	Mean ± SD	88 ± 0.3	70 ± 0.09	77 ± 0.1	68 ± 0.11	69 ± 0.17
	*p*-value	----------	0.043 *	0.241	0.055	0.033 *
*Rhodotorula* sp.	Mean ± SD	87.7 ± 0.7	72.9 ± 0.1	78 ± 0.09	70 ± 0.16	67 ± 0.1
	*p*-value	----------	0.025 *	0.286	0.109	0.055
*C. famata*	Mean ± SD	88 ± 0.3	70 ± 0.55	75 ± 0.11	66 ± 0.1	65 ± 0.08
	*p*-value	----------	0.014 *	0.166	0.055	0.019 *

* Significant at *p* < 0.05.

## Data Availability

All data are presented in the current study. Raw data are available on request from the corresponding author.

## Supplementary Materials

The following supporting information can be downloaded at: https://www.mdpi.com/article/10.3390/nano13152198/s1, Figure S1: Schematic diagram showing the synthesis process of silver nanoparticles from the aqueous extract of *C. citratus*.
