# Supplementary material for: Biological Activity of Biosynthesized Silver Nanoaggregates Prepared from the Aqueous Extract of Cymbopogon citratus against Candida spp
Source: Nanomaterials (Basel). 2023 Jul 28;13(15):2198. doi: 10.3390/nano13152198 (PMC10421022; doi:10.3390/nano13152198)
Supplement: Supplementary file 1 [file nanomaterials-13-02198-s001.zip › nanomaterials-2473125-supplementary.pdf]

nanomaterials

Supplementary Materials

Biological Activity of Biosynthesized Silver Nanoaggregates Prepared from the Aqueous Extract of *Cymbopogon citratus* against *Candida* spp.

Fatimah Al-Otibi *, Luluwah S. Albulayhid, Raedah I. Alharbi, Atheer A. Almohsen and Ghada M. AlShowiman

Department of Botany and Microbiology, College of Science, King Saud University, Riyadh 11495, Saudi Arabia; lulusul09@gmail.com (L.S.A.); raalharbi@ksu.edu.sa (R.I.A.); atheer.ab.almohsen@gmail.com (A.A.A.); gshowinan@ksu.edu.sa (G.M.A.)

* Correspondence: falotibi@ksu.edu.sa; Tel.: +966-11-805-5970

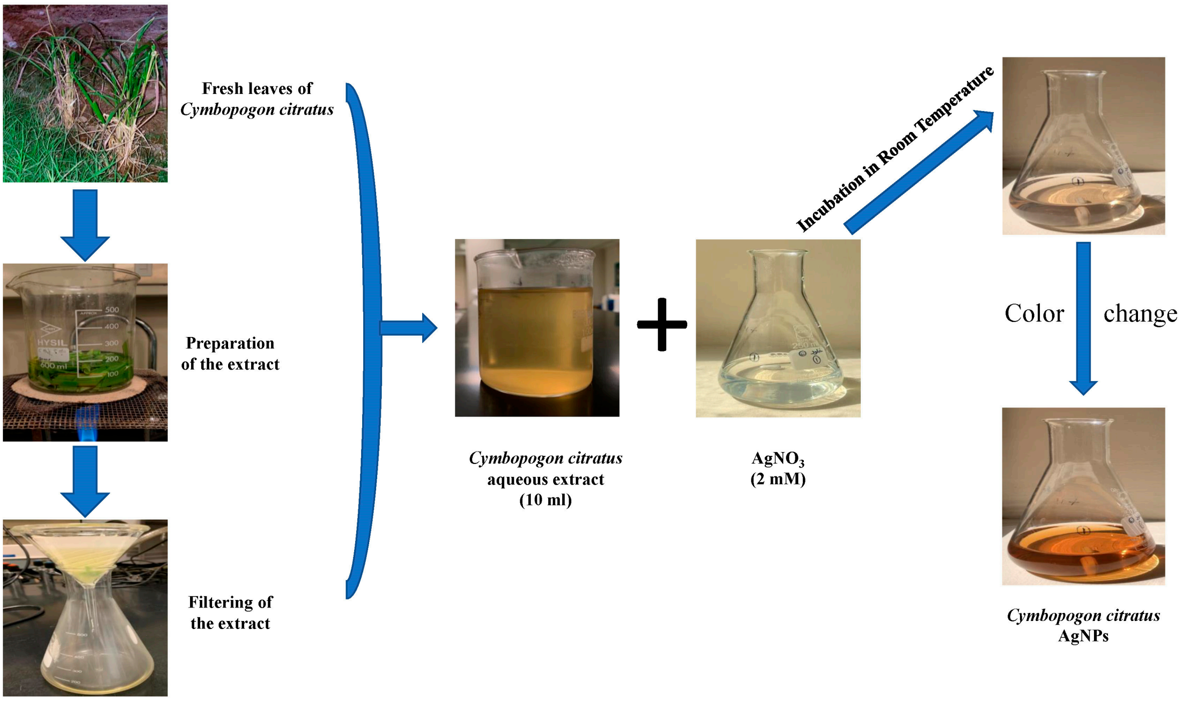

Figure S1. Schematic diagram showing the synthesis process of silver na-noparticles from the aqueous extract of *C. citratus.*
